# Effects of Scale, Question Location, Order of Response Alternatives, and Season on Self-Reported Noise Annoyance Using ICBEN Scales: A Field Experiment

**DOI:** 10.3390/ijerph13111163

**Published:** 2016-11-23

**Authors:** Mark Brink, Dirk Schreckenberg, Danielle Vienneau, Christian Cajochen, Jean-Marc Wunderli, Nicole Probst-Hensch, Martin Röösli

**Affiliations:** 1 Federal Office for the Environment, CH-3003 Bern, Switzerland; 2 ZEUS GmbH, D-58093 Hagen, Germany; schreckenberg@zeusgmbh.de; 3 Swiss Tropical and Public Health Institute, CH-4002 Basel, Switzerland; danielle.vienneau@unibas.ch (D.V.); nicole.probst@unibas.ch (N.P.-H.); martin.roosli@unibas.ch (M.R.); 4 University of Basel, CH-4002 Basel, Switzerland; 5 Centre for Chronobiology, Psychiatric Hospital of the University of Basel, CH-4002 Basel, Switzerland; christian.cajochen@upkbs.ch; 6 Empa Swiss Federal Laboratories for Materials Science and Technology, CH-8600 Dübendorf, Switzerland; jean-marc.wunderli@empa.ch

**Keywords:** road traffic noise, noise annoyance, survey methodology, ICBEN scales, context effects, season, field experiment

## Abstract

The type of noise annoyance scale and aspects of its presentation such as response format or location within a questionnaire and other contextual factors may affect self-reported noise annoyance. By means of a balanced experimental design, the effect of type of annoyance question and corresponding scale (5-point verbal vs. 11-point numerical ICBEN (International Commission on Biological Effects of Noise) scale), presentation order of scale points (ascending vs. descending), question location (early vs. late within the questionnaire), and survey season (autumn vs. spring) on reported road traffic noise annoyance was investigated in a postal survey with a stratified random sample of 2386 Swiss residents. Our results showed that early appearance of annoyance questions was significantly associated with higher annoyance scores. Questionnaires filled out in autumn were associated with a significantly higher annoyance rating than in the springtime. No effect was found for the order of response alternatives. Standardized average annoyance scores were slightly higher using the 11-point numerical scale whereas the percentage of highly annoyed respondents was higher based on the 5-point scale, using common cutoff points. In conclusion, placement and presentation of annoyance questions within a questionnaire, as well as the time of the year a survey is carried out, have small but demonstrable effects on the degree of self-reported noise annoyance.

## 1. Introduction

The reaction or emotion of being annoyed by noise can hardly be observed in a person directly, but must be assessed via self-reports. Yet the responses to annoyance questions in a survey may easily be affected by the methodological approach itself, e.g., by the way the noise topic is presented, and by factors such as the sequencing of annoyance questions, the wording of a question, the choice and order of response alternatives, and many others. Small changes in design, wording, item positioning, but also survey context or season may introduce variance of annoyance ratings which compromises the reliability and comparability of findings [1,2,3,4]. Owing to this, the research on survey context in the noise annoyance domain [5,6] and research about effects of the annoyance measurement scale employed [7,8] has recently gathered momentum.

In 2001, the International Commission on Biological Effects of Noise (ICBEN) published standardized noise annoyance scales and corresponding question stems that are intended to yield internationally comparable measures of annoyance reactions in noise surveys [9]. To measure annoyance intensity of residents that are affected by noise, ICBEN recommended the use of two different scales: the (so called) 5-point verbal and the 11-point numerical annoyance scale. The recommendation for the question wording—and specifically the scale point labels—was the result of a standardized empirical study protocol that has been applied in several sub-studies carried out in different languages and countries. While the ICBEN recommendation specifies the wording of questionnaire items (question stem) and labeling of scale points, other aspects of the presentation of the two questions in a questionnaire or interview are not explicitly addressed, such as order of response alternatives, question sequence, or effects of the season in which a noise survey is carried out. Back in 2001, the ICBEN steered clear of suggesting a “preferred” interview mode and also left it to future research to study the variability of annoyance assessment that may arise due to subtle differences of the application of their annoyance scales in noise surveys.

Some of the potential effects of survey design characteristics on reported noise annoyance shall be briefly discussed:

- Type of contact: The ICBEN recommendation states that both annoyance scales can be used irrespective of the type of contact (either personal, self-administered/by postal mail, or via telephone).
- Type of question and scale: While the numeric 11-point scale is believed to provide greater assurance that the scale points are equally spaced and hence be suitable for linear regression analysis, the 5-point verbal scale is suggested to be used when communication between respondents and policy makers stands in the foreground [9]. Nevertheless, in any given study, it is up to the researcher to decide upon which question’s results (5-point or 11-point) to finally use for the formulation of exposure–response relationships for annoyance as well as for the percentage of highly annoyed persons (%HA) that are regarded as the “valid” outcome of the study. ICBEN recommends to always use both questions. Still, it has not been investigated so far whether the particular sequence of the presentation of the two questions (5-point question first, then 11-point question, or vice-versa) affects the answering behavior.
- Order of response alternatives (scale points): In psychological method studies, the order in which response alternatives are presented has been shown to influence the answering behavior of respondents [10]. While the ICBEN recommendation for the 5-point verbal scale is to present it with the highest intensity (“extremely”) at the top/left, the corresponding International Organization for Standardization (ISO) standard [11] recommends the opposite, namely to put the lowest intensity (“not at all”) at the top/left of the scale. On the same note, in order to save space on a self-administered questionnaire, the 5-point verbal question/scale is usually not presented vertically as recommended both by ICBEN and ISO, but in a horizontal manner. It is yet unknown if and to which degree such differences in question presentation affect the response behavior.
- Location/placement of annoyance questions: The place where questions appear in a questionnaire can affect the way in which respondents interpret and thus answer them [10,12]. ICBEN generally recommends placing the annoyance questions early in the inter-view/questionnaire. However, to our knowledge, studies to empirically test the effect of the location of ICBEN annoyance questions have not been carried out yet. For the researcher, it is certainly important to know if annoyance questions in a questionnaire (which may contain many other noise-related questions) are treated differently by the respondents, depending on whether the annoyance questions are asked after the respondent filled out a range of other noise-related questions, or before such questions.
- Season: There is no clearly preferable time of the year for noise annoyance surveys to be carried out in order to inform general noise policy decisions, but surveys are seldom carried out in the middle of the winter or during the peak period of the holiday season in summer. In most general noise surveys, the idea is that respondents rate their average long-term noise annoyance, i.e., in the preceding one-year period. This also finds expression in the aforementioned ICBEN standard questions that both begin with “Thinking about the last 12 months...”. However, despite this clear instruction, meteorological and maybe also other non-weather but time of the year-related circumstances may still affect annoyance responses at the time of a survey. The available evidence of seasonal effects have been analyzed by Miedema et al. [2], who suggest that previous studies’ estimates of long-term noise annoyance reactions may have been affected by the time of the year when residents were interviewed, with higher annoyance in warmer seasons.

In order to systematically evaluate differences in the annoyance responses between the 5-point and 11-point ICBEN scales as a function of their presentation in a postal questionnaire, we carried out a socioacoustic survey within a stratified random sample of road traffic noise-exposed inhabitants in German-speaking Switzerland. We investigated to which degree the type of question/scale (5-point vs. 11-point), sequence of questions (5-point → 11-point vs. 11-point → 5-point), order of the scale’s response alternatives (ascending vs. descending), question location within the questionnaire (early vs. late), and season during which the survey was carried out (here, “autumn” vs. “spring”) affected annoyance responses.

## 2. Methods

### 2.1. Overview

By means of a balanced factorial design (detailed below) in a postal survey, we investigated the research questions stated above. To consider the effect of season, the survey was carried out in two waves during two different times of the year with 1220 and 1211 questionnaires returned, respectively. In the first wave, in the year 2012, questionnaires were sent out on 9 October, and were collected until the end of November. In the second wave, questionnaires were sent out on 8 March 2013, and were collected until end of April. Thus, the season that just preceded the survey was late summer/early autumn in the first wave, and late winter/early spring in the second. No follow-up mailings (e.g., reminder cards) were mailed out.

### 2.2. Survey Design

#### 2.2.1. Sample Size Estimation

Sample size estimation for the present study was carried out using the R software version 3.01 (R Development Core Team, Vienna, Austria) by bootstrap resampling from two previously obtained datasets, i.e., from a survey on aircraft noise annoyance (*n* = 2269) in Frankfurt [13] and from a study on military shooting noise annoyance (*n* = 1002) around training grounds of the Swiss army [14]. Both these datasets contained ICBEN annoyance ratings for both the 5-point and 11-point scale. The parameter in the focus of sample size estimation was a 5% difference in the R-square value between linear models that use one or the other scale to measure annoyance. The analysis revealed the minimal required power 0.8 to be reached at a sample size of about *n* = 500 based on the aircraft noise data, and about *n* = 850 based on the military shooting noise data.

#### 2.2.2. Sampling Strategy

Assuming a response rate of at least 25%, we posted 2 × 4200 short questionnaires, with a cover letter, to randomly selected road traffic noise-exposed residents in the German-speaking part of Switzerland (here, defined as the set of cantons where German is the predominantly spoken language: Aargau, Appenzell, Basel, Bern, Glarus, Graubünden, Luzern, Nidwalden, Obwalden, Schaffhausen, Schwyz, Solothurn, St. Gallen, Thurgau, Uri, Zug and Zürich), according to the factorial design described below. The survey was conducted in accordance with the Declaration of Helsinki. Ethical approval for the conduct of the survey was secured from the Ethics Commission of ETH Zurich (the institution where the study was carried out) in 2012. No compensation was paid for participation.

#### 2.2.3. Noise Exposure Assessment

Road traffic noise exposure assessment was carried out based on the latest release of sonBASE, the Swiss national noise monitoring database [15]. In addition to spatial geoinformation and exposure data for the sources road traffic, railways, and aircraft, sonBASE integrates building address data and population figures from official registers in a homogeneous geographical information system (GIS). Each building structure in sonBASE is modelled with three facade points per facade and floor, larger building complexes may thus be modelled with hundreds of receiver points. From the entire German-speaking part of Switzerland, we randomly selected residential buildings from sonBASE records assigned to seven 2.5 dB-wide exposure categories between 52.5 dB(A) and 70 dB(A) Ldn (Day–Night Level) of road traffic noise, according to the most exposed facade point at mid-height of the building. The selected building records were matched to census data provided by the Swiss Federal Statistical Office, yielding name, age, and gender attributes of the inhabitants. This allowed for the establishment of a stratified random sample of named individuals. After the questionnaires were returned, individual road traffic noise exposure was reassessed based on the floor information given by the respondents. This implicated that the overall exposure range in the sample increased from 44.3 to 77.5 dB(A) Ldn of road traffic noise exposure.

#### 2.2.4. Factorial Design

In the factorial design we applied two different survey seasons (“autumn” vs. “spring”, see Section 2.1), two sequences of annoyance questions (either the 5-point question or 11-point question was asked first), two orders of response alternatives (ascending vs. descending) and two question locations within the questionnaire (early vs. late), resulting in a 16 cell (2 × 2 × 2 × 2) between-subjects design, using eight different design variants (“A”–“H”) of the questionnaire (Table 1). Each of these cells were stratified into seven noise exposure (Ldn) categories. Seventy-five individuals were randomly assigned per exposure category per cell from the stratified sampling frame. The variable “scale type” is a within-subject factor in this design (as all questionnaire variants contained both ICBEN questions/scales).

#### 2.2.5. Meteorological Data

Systematically collected weather data were obtained to characterize the meteorological conditions for each respondent during the two survey periods as well as to control for potential weather effects in the exposure-effect models. Average day and night temperature, daily precipitation, and sunshine hours per day at the time of filling out the questionnaire and during the days and weeks before were assigned to each respondent based on the respondent’s nearest weather station operated by the Federal Office of Meteorology and Climatology MeteoSwiss. Average weather statistics were calculated for each respondent for periods of 30 days and 7 days before, and on the day of completing the questionnaire. 

#### 2.2.6. Questionnaire

For the survey, a two-sided single sheet questionnaire was designed. It was deliberately kept short, thus less time-consuming to fill out, in order to try to maximize the response rate. The survey was entitled “Short survey on noise in the living environment” and was mailed together with an accompanying letter that stated the funding source and research institution. A postpaid envelope for returning the questionnaire by mail was enclosed. A total of eight variants (“A”–“H”) of the questionnaire were produced, which varied only by the annoyance questions location, sequencing of the annoyance questions, and order of response alternatives, in order to match the factorial design as given in Table 1. The questionnaire can be viewed in two variants (“A” and “H”), in the original German language (GER) and with the English translation (ENG) in the Supplementary Materials.

In the header of the questionnaire, the respondents were explicitly instructed to answer the questions sequentially, starting with the first one (German: “der Reihe nach”). All questionnaire variants started with a few general questions at the beginning, which delivered the following variables: age, sex, marital status, duration of residency, household size, type of dwelling and ownership of house/apartment. After this first block, in the “early” condition (Variant “A” questionnaires; see Supplementary Files), the two annoyance questions were asked. The remainder of the questionnaire was made up of questions about the satisfaction with different aspects of the living environment (e.g., size of dwelling, distance to work, lightness of the dwelling and noise exposure), questions about situation-specific disturbances by road traffic noise (e.g., “disturbs conversations, phone calls”, “disturbs when being outside”, “disturbs at night when I want to sleep” etc.), and whether the respondent is specifically disturbed by certain vehicles on the road (cars, trucks, motorbikes, trams and buses), and at specific time periods during the day. Then, a single 11-point numerical scale followed, which measured general noise sensitivity. The next questions were devoted to sleeping habits, ventilation of the sleeping room, and window opening behavior during summer and winter. The following question asked the respondents if they used earplugs for sleeping and if yes, for which reasons. Finally, respondents filled in the actual date. In the “late” condition Variant ‘H’ questionnaires (see Supplementary Files), the annoyance questions were asked at the end of the questionnaire.

The degree of road traffic noise annoyance was assessed by both the 5-point verbal ICBEN scale with the point labels “not at all”, “slightly”, “moderately”, “very”, “extremely”, and the 11-point numerical scale, either placed at the beginning (“early” condition) or at the end (“late” condition) of the questionnaire, both in a horizontal fashion. The question stems were formulated according to the ICBEN recommendation ([9], p. 651), but were slightly adapted for the paper-and-pencil form and written in past tense, namely, for the 5-point verbal scale, in English: “Thinking about the last 12 months, when you are here at home, how much did road traffic noise bother, disturb, or annoy you?”; and for the 11-point numerical scale “Below is a 0 to 10 opinion scale for how much road traffic noise bothered, disturbed or annoyed you. If you were not at all annoyed choose 0, if you were extremely annoyed choose 10, if you were somewhere in between choose a number between 0 and 10. Thinking about the last 12 months, what number from 0 to 10 best shows how much you were bothered, disturbed, or annoyed by road traffic noise?’’.

It was deemed that the order of response alternatives (either from low to high intensity, or high to low intensity) should be identical for both questions so as to not confuse respondents.

### 2.3. Statistical Analysis

#### 2.3.1. Scale Conversions

To carry out absolute numerical comparisons between the two annoyance questions and corresponding scales, the 5-point and the 11-point scales were linearly transformed to a numeric absolute scale ranging from 0 to 100 by assuming the original scales to be equidistant interval scales and that the first and last scale point on the 5-point scale (“not at all” and “extremely”) and the scale points “0” and “10” on the 11-point scale represent the endpoints of the same annoyance intensity continuum that ranges between minimal (i.e., inexistent) and maximum (i.e., unbearable, extreme) annoyance (Table 2). Such a linear transformation appears justified as equidistance between the scale points was one of the primary goals in the development of the 5-point ICBEN scale.

Nevertheless, the “correct” rule of transforming the original scale point labels to values between 0 and 100 is not inherently obvious, because it is basically unknown whether respondents interpret the point labels as a descriptor of a discrete point or as a midpoint (or lower or upper boundary for that matter) of a category that occupies an equal amount of the scale’s total length. For the 11-point numerical scale from 0 to 10, equidistance and linearity may be taken for granted, hence a linear upscaling to a larger range may not pose any problems. However, some issues surrounding the choice of a numerical equivalent for scale point labels on the 5-point verbal scale have not been fully resolved; for a discussion see Fields et al. [9]. While Miedema and Vos popularized the “midpoint conversion” [16], we use the “discrete point conversion”, as tabulated in Table 2 above, in the analyses and comparisons in this paper. This kind of conversion is chosen for several reasons: Basically, multiplying the scale value on the 11-point scale by a factor of 10 provides the simplest and most immediately understandable upscaling to the range 0–100. Multiplying by 25 also allows upscaling of the numeric values of the 5-point scale (as given in Table 2) to values expressible as integers while not only preserving equidistance between scale points, but also preserving the zero (0) anchor point (that has a conceptually unambiguous meaning and should clearly express “not annoyed at all”). However, the discrete point conversion may overestimate the annoyance intensity slightly at the highest point (“extremely”, 100) as probably not all “extremely” annoyed people would put themselves at exactly 100 on an underlying 0–100 intensity scale. As will be shown later (see Figure 2a in the Results section), the average intensity on the 11-point scale, ranging from 0 to 10, of respondents reporting to be “extremely” annoyed in the present study was in fact 9.5, not 10. Implications of this observation are discussed later.

#### 2.3.2. Analysis of Agreement and Statistical Modeling

To assess whether the two scales could be used interchangeably by a given standard of accuracy, the “limits of agreement” between the annoyance score obtained by the 5-point and 11-point scale were calculated—as suggested by Bland and Altman [17]—by comparing the difference of scores between the two measurement scales against the mean of both scores and by constructing a confidence interval for the mean difference.

The effects of annoyance scale and question presentation characteristics on the (discrete point) annoyance score and the probability of high annoyance (P_HA_) were modeled with the repeated measures linear mixed model and logistic mixed model as implemented in the MIXED and GENLIN procedures of IBM SPSS Statistics version 20 (IBM Corp., 2011, Armonk, NY, USA). “High annoyance” was assigned to all cases with the answers “very” and “extremely” on the 5-point scale (following the original ICBEN recommendation) and values greater or equal than 8 on the 11-point scale respectively (following widespread convention), corresponding to cutoff values 40% and 28% of the total scale length. Generally, an alpha level of significance of 0.05 was assumed.

## 3. Results

In the following, results are reported in five parts: In Section 3.1, response statistics and sample characteristics are described; in Section 3.2, exposure-annoyance relationships using both scales are presented; in Section 3.3 we report about the degree of agreement between the two scales; Section 3.4 deals with possible conversions between the two scales; and in Section 3.5 the effects of season, scale and question presentation characteristics on annoyance responses are investigated.

### 3.1. Response Statistics and Sample Characteristics

In Wave 1 (autumn), from a total of 4200 persons individually addressed, 1220 completed one-sheet questionnaires were sent back. The return rate was very similar in Wave 2 (spring), with 1211 questionnaires sent back. In the first wave, 19 questionnaires were excluded and 26 were excluded in the second because respondents indicated that they did not permanently live at the address for which the noise exposure was calculated.

Overall response statistics are given in brief in Table 3. The cooperation rate is referred to as the proportion of returned non-empty questionnaires from the initial mail-out of 8400 questionnaires. However, the usable number of cases is somewhat smaller: From the 2386 returned non-empty questionnaires, there were eight missing answers in the 5-point question and 25 missing in the 11-point question. Finally, this gave a rate of usable responses (response rate) of 0.28. In both waves, about 50% of the returned questionnaires were filled out within 4 days, and 75% within 7 days after we sent them out. The latter corresponds to the fill-out periods of 10–16 October (autumn wave) and 11–17 March (spring wave).

Sample characteristics in each (recalculated) exposure category are given in Table 4. Exposure accounted for the loudest facade point on the floor where respondents reported their apartment/dwelling was located. Across both waves, respondents were estimated to be exposed to road traffic noise levels of between 44.3 and 77.5 dB(A) Ldn. We tested for the average difference in road traffic noise exposure between the two waves, but could not find any significant difference for any of the metrics Ldn, L24h, LDay, or LNight.

The two survey waves (seasons) differed, as expected, in several meteorological parameters which are tabulated in Table 5. The table shows average weather parameters that were individually calculated for each respondent based on his/her nearest MeteoSwiss weather station and averaged over the periods of 30 days and 7 days prior to the day the questionnaire was filled out, and on the day the questionnaire was filled out. As expected, the temperature difference between the two seasons was larger for longer averaging periods. The average day temperature in the autumn wave in the 30 days prior to completion of the questionnaire was about 12 degrees Celsius higher than in the spring wave.

### 3.2. Exposure-Effect Relationships

**Figure 1:**
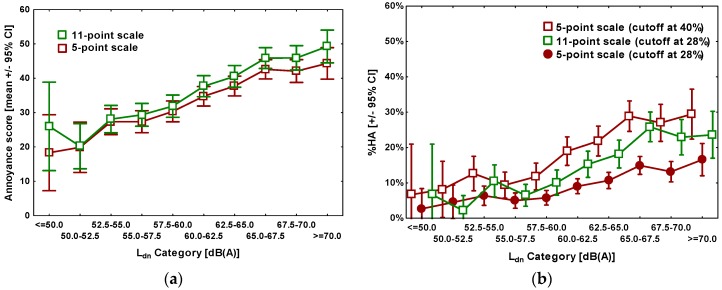
Exposure-effect diagrams. (**a**) Mean annoyance score (discrete conversion value, including ±95% confidence intervals (CIs)) by exposure category; (**b**) Percentage highly annoyed (%HA, including ±95% CIs) by exposure category (for better visibility, data series are slightly shifted).

Figure 1 shows mean annoyance ratings (Figure 1a) as well as the percentage highly annoyed (%HA) (Figure 1b) measured with both scales, plotted against Ldn. In total, on the 5-point verbal scale 481 of 2386 respondents qualified as “highly annoyed”, and 398 on the 11-point numerical scale, according to the normal cutoff criteria.

To account for the normally used different cutoff points of the 11-point and the 5-point scale in the calculation of HA, the HA-percentages for the 5-point scale were calculated in two ways, firstly using the conventional cutoff point at 40% of the scale length, corresponding to the two uppermost scale points (“very” and “extremely”), as recommended by ICBEN [9], and secondly, mimicking a cutoff point of 28% by weighting the answer alternative “very” with a factor of 0.4, according to the principle set forth by Miedema and Vos [16].

Basically, as can be seen in Figure 1a, the differences between the average annoyance score ratings obtained by the two scales are rather small (between about 2 and 8 points within the possible range of 0–100 points). While the ratings on the 11-point scale are somewhat higher, one can still conclude that on average, the two scales can be considered to yield very similar annoyance scores within given exposure categories. Things look slightly different with the %HA measure: In all exposure categories, %HA was higher based on responses on the 5-point scale than on the 11-point scale. This was of course a well-expected result, due to the larger cutoff value on the 5-point scale (at 40%). However, weighting the answers on the 5-point scale according to the same cutoff as adopted for the 11-point scale (28%), puts the %HA considerably below the corresponding value on the 11-point scale in almost all but the lowest Ldn categories. It becomes obvious that even using the widespread approach of attaching weights to scale points to align cutoff points on the two different annoyance rating scales does not automatically produce congruent exposure-annoyance relationships.

### 3.3. Degree of Agreement between the Two Scales

**Figure 2:**
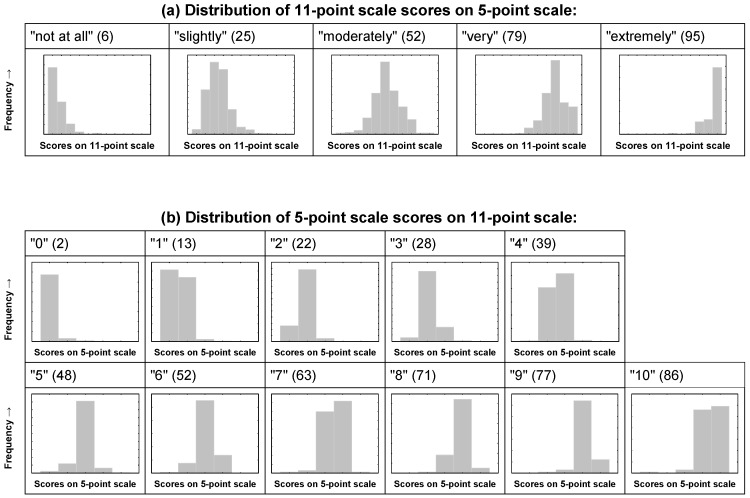
Distribution of annoyance score values. (**a**) Distributions of absolute frequencies of the annoyance score on the 11-point scale for each answer (“not at all”, “slightly”, “moderately”, “very” and “extremely”) on the 5-point scale; (**b**) Distributions of absolute frequencies of the annoyance score on the 5-point scale for each score on the 11-point scale. The figures in brackets represent the mean score (discrete conversion value) on the respective other scale, rounded to the nearest integer.

In Figure 2 below, the frequency distributions of the chosen response alternatives on one scale are shown for each scale point on the respective other scale. Figure 2a shows the distribution of the answers on the 11-point scale for each scale point of the 5-point scale, and Figure 2b does so vice-versa. The figures in brackets represent the mean score (discrete conversion value, Table 2) on the respective other scale, i.e., the average value for the rating on one scale, given the rating on the other in the present sample (for example, the mean 11-point score given by respondents that chose the “very” scale point on the 5-point scale, is 79).

If one takes the empirical values on the upscaled 11-point scale as the reference to describe the “true” average annoyance intensity for each of the annoyance modifier words “not at all”, “slightly”, “moderately”, “very”, and “extremely”, Figure 2 should reveal if the discrete point conversion approach or the midpoint conversion approach is more suitable to align the two scales. Assuming a perfectly linear association between the two scales, we would expect the mean discrete conversion value of the 11-point score to be 0 for the “not at all”, 25 for the “slightly”, 50 for the “moderately”, 75 for the “very” and 100 for the “extremely” annoyed respondents. Similarly, we would expect the midpoint conversion values of the “not at all”, “slightly”, “moderately”, “very”, and “extremely” annoyed to be 10, 30, 50, 70, and 90, respectively. As we can see, the empirical mean values are 6, 25, 52, 79, and 95. This clearly shows that the discrete point conversion worked quite well for the three scale points in the middle of the scale not marking the extremes, but that the discrete point conversion underestimates the annoyance intensity at the lower end (0 instead of 6), and overestimates the intensity at the upper end of the 5-point scale (100 instead of 95). In contrast, the midpoint conversion reflects annoyance intensity slightly better at the lower end of the scale, however it still overestimates it (10 instead of 6). At the same time, the midpoint conversion underestimates the intensity at the upper end of the scale (90 instead of 95) by the same amount (5 points) as the discrete point conversion overestimates it. All in all, there is no clear evidence that one conversion method would markedly outperform the other with regard to the extremes of the scale. Regarding the three middle points on the 5-point scale, the discrete point conversion seems to (a bit more accurately) capture the intensity reflected in the 11-point scale. Therefore, it was decided to retain the discrete point conversion for the purposes in this paper.

**Figure 3:**
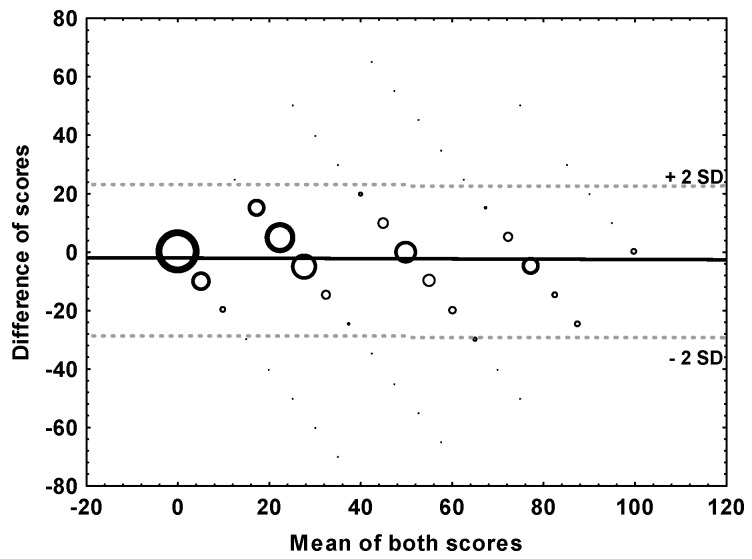
Bland-Altman bubble plot of the difference of the annoyance score (mean difference = −2.6, solid line), versus their mean including limits of agreement, as indicated by ±2 standard deviations (SDs) of the difference (dashed lines). Note: Bubble size is proportional to the frequency of responses having the particular combination of mean and difference; *N* = 2378.

Figure 3 shows a Bland-Altman [17] bubble plot for the difference between the two scale ratings (as expressed in the discrete conversion value) against their mean. For the “limits of agreement”, defined here as the range of differences within ±2 standard deviations from the mean difference (which is −2.6 points), the lower and upper bounds were −29 and 24 points, respectively. This means that 95% of the individual differences in score values fell within these boundaries.

### 3.4. Simple Conversion between the Values on Both Scales

One might want to estimate the average score value on one scale given the average value on the other, using a simple conversion/transformation rule, e.g., for combining values on the two scales for the purpose of meta-analysis. By using the data set in this study, this can either be achieved by (a) employing linear regression to estimate the value on one scale, regressed on the value of the other; or (b) by simply considering the empirical mean intensity discrete point conversion scores, as they are given in brackets in Figure 2.

**Figure 4:**
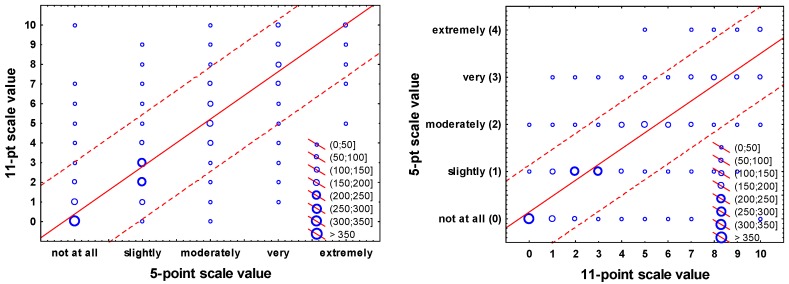
Frequency scatter plot of the annoyance rating on the 11-point scale as a function of the 5-point scale and vice-versa, including prediction interval.

In Figure 4 below, the frequency distributions of the chosen response alternatives on one scale, given the values on the other, are shown for each scale point, including linear regression line and prediction interval. The equations for the linear conversion between the numeric scale values (0–4 for the 5-point scale, and 0–11 for the 11-point scale) are, using regression with all data points: 11-point scale value = 0.3861 + 2.4135 × 05-point scale value; 05-point scale value = 0.1422 + 0.3361 × 11-point scale value. We are aware that these are relatively simple “rules” that certainly call for a more thorough modelling approach, including population estimates of the confidence of conversions, but we believe that such an analysis should be carried out using a much larger data set with data from multiple studies, different languages, and additional noise sources.

### 3.5. Effects of Season, Scale Type and Question Presentation Characteristics on Reported Annoyance

#### 3.5.1. Effects on Annoyance Score

In a first step, to test the potential effect of weather conditions during the weeks and days before and on the very day the questionnaire was filled out, on annoyance (discrete point conversion), a series of repeated measures linear mixed models were calculated incorporating the following meteorological parameters:

- average day temperature in the period 05:40–17:40 h (°C).
- total precipitation in the period 07:00–19:00 h (mm).
- absolute number of sunshine hours within the 24-h period (h).

Each of these parameters was entered into separate models together with type of scale, sequence, order of response alternatives, location (of scale) in questionnaire, and the covariate noise exposure, as either the 30, 7, or 1-day individual average in the period before the date the questionnaire was filled out. Season itself was omitted as a factor in these models to avoid collinearity with the weather variables.

The only significant, but very small effect found was for the day air temperature average over 30 preceding days: An increase of 1 °C in air temperature increased the discrete point conversion value of the annoyance score by the amount of 0.16 points. This effect is very small, but its direction is in line with previous reports [2]. In the statistical models that are developed in the following, weather data are not accounted for.

We analyzed differences in the discrete point annoyance score yielded by the two scales by a repeated measures mixed model, modeling the effect of type of scale as a within-subject (repeated), and the season, sequence, order of response alternatives, and location as between-subject factors, with noise exposure as a covariate. After observing that there were no significant two-way interaction effects between the type of scale and any of the other factors, the final model contained all main effects, but no interaction terms. The coefficients of the final model are tabulated in Table 6.

Besides the expected significant effect of the road traffic noise exposure, a significant question/scale effect could be observed, with the 11-point scale yielding 2.76 points (discrete conversion value) higher annoyance scores. The season of the survey also had an effect on the annoyance rating, as hypothesized earlier [2], with on average 2.10 points higher annoyance scores in autumn (survey carried out October/November) than in spring (survey carried out March/April). The location of the annoyance item in the questionnaire showed a significant effect on the annoyance score as well: annoyance questions appearing early in the questionnaire resulted in annoyance scores 4.18 points higher. The factors sequencing of the annoyance questions and order of response alternatives showed no significant effects.

Effects of the independent predictor variables on the annoyance score can also be expressed in decibel values, as the ratio of coefficients. In the present study, using the 11-point scale instead of the 5-point scale increased the annoyance score by the same amount as a 2.2 dB increase in Ldn (2.763/1.272); average annoyance responses during the autumn wave were increased by the same amount as a 1.7 dB increase in Ldn, as compared to early spring. Asking about annoyance early in the questionnaire had the same effect on the annoyance score as an increase of 3.3 dB in Ldn.

**Figure 5:**
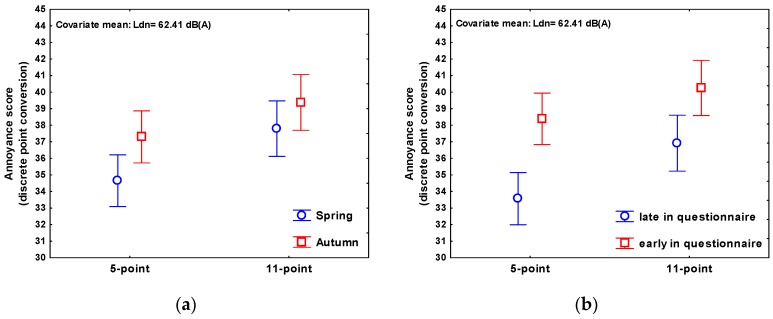
Least squares means plots. (**a**) Annoyance score (discrete point conversion) by season, as measured by the 5-point and 11-point scales, ±95% CI; (**b**) Annoyance score (discrete point conversion) by location of annoyance questions, as measured by the 5-point and 11-point scales, ±95% CI.

Least squares means profile plots of the annoyance score on both scales by season and by location (adjusted for the other predictors in the model) are given in Figure 5.

The amount of variance of annoyance explained by the predictors in the model was rather low, with an adjusted R-square value of just 0.07. However, it is important to note that the above model is based on the individual response data and not on aggregate (average) measures of annoyance in distinct exposure categories. With individual data, normally, bivariate exposure-annoyance models reach R-squared values between about 0.05 and 0.25, as was reported in a recent review [18]. Given that, R-squared is still low, but not atypically low.

#### 3.5.2. Effects on the Probability to Be “Highly Annoyed” (P_HA_)

Similar to the linear model described in the preceding section, the probability of being “highly annoyed” (P_HA_) was regressed on season, type of scale, sequencing of the annoyance questions, order of response alternatives, location and the exposure variable Ldn, by means of a repeated measures logistic regression analysis. Since we did not observe any significant two-way interaction effects between the type of scale and any of the other factors, the final model contained all main effects, but no interaction terms. Results are tabulated in Table 7. As the results show, in contrast to the annoyance raw score, the probability of being highly annoyed is not significantly associated with season. The effect of scale type is significant, most probably due to different cutoff values used for the “highly annoyed” assignment. The presentation of the annoyance questions early in the questionnaire increased the likelihood of reporting to be highly annoyed significantly, by a factor of 1.28.

**Figure 6:**
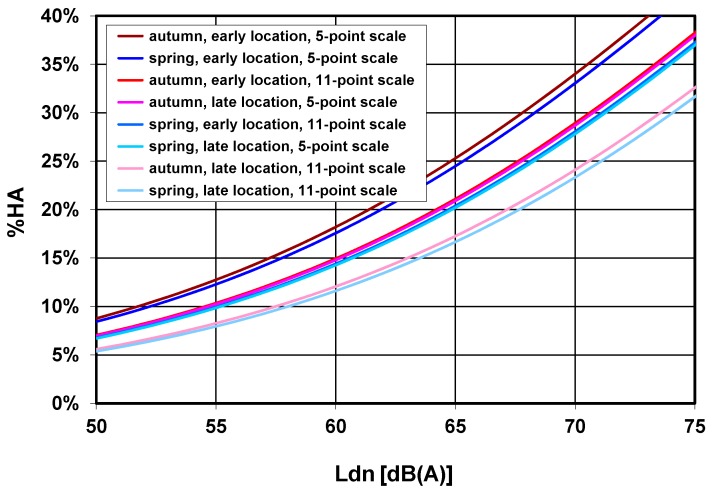
Percentage highly annoyed (%HA, using a 40% (5-point scale) and 28% (11-point scale) cutoff point, respectively) as a function of Ldn and various factor combinations, based on coefficients of the logistic model in Table 7. The factors “Sequence of annoyance question” and ”Order of response alternatives” were kept constant in the plot and set to “5-point → 11-point” and “ascending” respectively. Note: for better readability, the series order matches the level of %HA.

Based on the unstandardized regression (B) coefficients given in Table 7, Figure 6 displays the logistic exposure-response curves for different factor combinations (for better visibility omitting confidence bands).

Figure 6 shows marked shifts of the exposure-response curve, depending on the particular factor combination applied. One can observe three distinguishable groups of function curves: The highest percentages of %HA are associated with the 5-point scale and an early location of the annoyance question in the survey, as compared to the two curves with the lowest overall percentages, which are the result of the use of the 11-point scale and late location of annoyance questions.

## 4. Discussion

### 4.1. Brief Summary

The present study aimed at elucidating differences in annoyance responses as determined by the use of either the 5-point verbal or 11-point numerical ICBEN scale and their presentation characteristics in a postal paper-and-pencil questionnaire about road traffic noise annoyance. By means of a balanced experimental design we investigated the effect of type of scale (5-point verbal vs. 11-point numerical) and sequencing of the annoyance questions, presentation order of response alternatives (ascending vs. descending), the location (placement) of noise annoyance questions within the questionnaire (early vs. late), and the time of the year, here, referred to as “season”, in which the survey was carried out. We also looked at the degree of agreement between the two scales in the measuring of annoyance intensity as well as at the accuracy of different conversion methods to align the two scales (midpoint vs. discrete point conversion).

Response rate in the survey was about average, with roughly 30% of the questionnaires sent back. We found that the two scales (5-point vs. 11-point) only slightly differed in the average annoyance score within a given exposure category. After standardization to a 0–100 scale, the 11-point scale elicited slightly higher annoyance score ratings than the 5-point scale (Figure 1). This is in line with findings of a review by Janssen et al. [8]. However, this was reversed in the context of logistic regression with “high annoyance” as the binary outcome: here, ratings using the 5-point scale (and a cutoff point at 40%) yielded higher percentages of highly annoyed respondents. Most importantly, depending on factor combination, we could observe marked shifts of the exposure-response curve for %HA up to about 6–7 dB (Figure 6).

Our study also made it clear that using the widespread approach of attaching weights to scale points (e.g., a weighting factor of 0.4 to the “very” scale point on the 5-point scale) to produce a “consistent cutoff” [16] (of 28%) does not automatically produce congruent relationships between exposure and %HA (Figure 1b). 

While for a given exposure category, the *average* (mean) annoyance ratings were very close to each other, the analysis of agreement on the *individual* level, as by the mean difference approach [17], yielded a quite wide interval (−29 to +24 points on a scale from 0–100) within which 95% of the differences in the annoyance ratings fell. It is up to the researcher´s individual judgement to decide whether such an interval is regarded as narrow enough to replace one scale by the other. We also determined how the annoyance intensity value on one scale can be estimated from the value on the respective other scale (measured in the same subject) and provided some simple conversion rules. There was no clear evidence that the “midpoint conversion”, as proposed by Miedema and Vos [16], captures the annoyance intensity on the 5-point scale any better than the “discrete point conversion” as defined in Table 2.

Given the present survey context, early appearance of annoyance questions in the questionnaire was positively associated with higher annoyance ratings as well as with a higher probability of reporting “high annoyance”. Consistent with the hypothesis that during warmer seasons, transportation noise is better audible in general, possibly because of different window opening behavior and more time spent outside, the survey wave carried out in autumn—right after the summertime—yielded higher annoyance ratings. We found no consistent evidence that the meteorological conditions on the day the questionnaire was filled out or during the immediately preceding days have any effect on reported long-term noise annoyance. However, we found a small effect on annoyance of the average air temperature in the preceding one-month period before the day questionnaires were filled out, with higher temperatures leading to slightly higher annoyance ratings, again, in agreement with common expectations. No significant effect could be found for the order of response alternatives and sequencing of the annoyance questions: we therefore conclude that both questions can be used alongside each other in a questionnaire if needed, and be arranged according to the requirements of a particular study.

### 4.2. Strengths and Limitations

The present study clearly has a number of strengths. Regarding the investigation of season effects, question placement, and order of response alternatives, this is, to our knowledge, the only survey that deliberately employed a balanced factorial design to systematically address the role of these factors in the measurement of annoyance responses using ICBEN [9] scales. While sequencing of the annoyance questions, order of response alternatives, and question location, including the covariate exposure, were varied as between-subject factors, the independent variable scale type constitutes a within-subject factor in the present design. It would, however, have been possible to also treat question location as a within-subject factor by repeating the two early annoyance questions at the end of the questionnaire, and vice-versa. We deemed this design alternative as problematic, since in practice, annoyance questions are usually only asked once in a questionnaire, and there would be no obvious explanation for the respondents as to why the same question was asked twice. This in turn could have provoked abnormal answering behavior (which was to be avoided). To determine the number of persons to interview, computationally intensive bootstrap resampling techniques were used to carry out power calculations. To estimate the expected response rate, the experience from previous research was considered. A clear advantage of the sampling procedure employed was that the sampling frame was based on official register data of individuals in the entire country, thus within each noise exposure stratum, a truly representative sample (of the German-speaking Swiss population) could be drawn.

We acknowledge that an important part of the results in the present paper is related to (and depending on) the conversion between the two scales, and this clearly relies on a linear assumption. The validity of this assumption might be questioned even if ICBEN’s original goal was to produce a 5-point scale with equidistant scale points, as evidenced in their reaction modifier study that preceded the formulation of their recommendations [9]. While we briefly evaluated the appropriateness of the midpoint and discrete point conversion methods to align the values on both scales (see Section 3.3), the inherent psychometric complexities of the issue cannot reasonably be addressed within the scope of this article, but should be the object of further research.

The sequence of the two annoyance questions did not show a significant effect on annoyance ratings. While there does not seem to be much of a reason for expecting the sequence in which the two questions appear to have any significant effect, it is legitimate to ask if the sequencing of annoyance questions affects the response behavior, especially as ICBEN recommends to always include both questions in a survey. However, the design of the present survey may not allow the conclusion that the sequence does not play a role if the 5-point verbal and 11-point numerical questions are posed wide apart from each other.

The present paper considers noise annoyance surveys with paper-and-pencil questionnaires. The differential effects of other interviewing modes (personal, telephone interviews, or online interviews) have not been investigated. It therefore remains to be determined whether the reported findings also apply for other interview modes and whether the choice of a particular interview mode modifies the effect of scale type and scale presentation characteristics.

The reported effect of the annoyance scale used to assess annoyance responses, though significant, must be put into perspective as explained variance in the present survey was overall rather low, with adjusted R-square values below 0.1. One explanation for the small R-squared values is the rather restricted exposure range employed in the present study, essentially excluding the road traffic noise exposed population below 50 dB(A) Ldn. This in turn reduces the achievable correlation between exposure and reaction.

The limitations regarding exposure range extend to the number of noise sources and exposure metrics the present findings can be generalized to. It remains to be demonstrated if the effects of annoyance question presentation as shown here, apply to other sources (railways and aircraft) also. Although potential interaction effects between exposure metric, source type and scale type cannot be excluded, the scientific literature so far does not provide a convincing a priori hypothesis which would suggest that other metrics or another source would yield a completely different result.

The topics addressed in the survey questionnaire and the number of items asked “between” the first and last question may determine the way respondents answer the noise annoyance questions at the end of the questionnaire. Thus one cannot be sure that the somewhat lower annoyance ratings in the “late” condition are just an effect of the late positioning of the annoyance questions within the questionnaire, or rather the result of the cognitive or emotional processing of the set of questions that had to be answered beforehand. It is well possible that some other aspect of the question location might have a bigger impact on response than the mere location at either the beginning or end of a questionnaire. Other survey techniques (e.g., personal or telephone interviews) with more control over which questions are asked (and answered) at what point in the interview, would probably be more suitable to assess the effect of an annoyance question being posed early or late. However, it seems unlikely that people started filling out the last question and then proceeded backwards, but unfortunately, this assumption cannot be verified. In the present survey, for the respondent, the questionnaire appeared to be clearly about noise and most of the questions asked had something to do with noise. The questionnaire was short, but contained many of the typical questions that are usually asked in noise annoyance surveys. Thus, we have confidence in the generalizability of our finding regarding the location effect, at least for shorter questionnaires or interviews.

The present selection of a few rather formal “questionnaire design-related” factors that may influence the response behavior is certainly not exhaustive. Important further sources of variability in the answering behavior in noise surveys are, more generally, context effects. For example, the findings of Kroesen et al. [6] support the hypothesis that measuring aircraft noise annoyance in relation to other noise sources creates a context in which people on average express a more extreme aircraft noise annoyance response. The criticism expressed by Brooker [19] about the methodology employed in the Attitudes to Noise from Aviation Sources in England (ANASE)-Study in the UK [20] also suggests that context is an important effect modifier in noise annoyance research. But as “context” is created by the entirety of the questions in a questionnaire and the way the issue is posed in the cover letter, it can hardly be captured in a manageable set of variables and thus can also hardly be fully controlled for. This limitation surely applies to the present, but would also apply to any other similar study.

One potential design flaw remains to be mentioned, concerning the investigation of the effect of “season” in our study. We estimated the effect of “season” in which two identical survey waves were carried out as a rather simple comparison between two arbitrary points in time that may not just simply differ by the calendar phase within any calendar year, but may also differ due to further time-related factors that we could not (and did not) control. Among them are effects of a (changing) general political climate, current affairs, or exigencies that may reduce or increase the relative importance given to the noise issue, or even influence annoyance ratings directly at the time of the two survey waves. This extends to the question if the two survey seasons truly represented a “typical” autumn or spring, e.g., in terms of (but not limited to) the average weather that prevailed during the survey periods. We are aware that such limitations restrict the generalizability of the season effect. Nevertheless, we observed an effect of season that was congruent with the general hypothesis, that annoyance ratings tend to be higher in warmer seasons. We also thoroughly objectified the differences of weather parameters to confirm the warmer weather during the autumn wave, compared to the spring wave.

Certainly, practical and logistical constraints aside, a more stringent design to estimate seasonal effects would mean distributing the mail-outs of questionnaires at regular small intervals (e.g., one month apart) across a prolonged period of, e.g., two full years. This would allow for quantification of the variability of annoyance responses between several time points within a single year (“between season variability”), and to compare it to the variability between years (“between survey variability”).

## 5. Conclusions

The placement and presentation of noise annoyance reaction questions, such as those proposed by ICBEN [9] within a short postal questionnaire, as well as the time of the year a survey is carried out, have small but demonstrable effects on the level of reported noise annoyance. Basically, our results show that one has to be aware of the differences in exposure-annoyance models that emerge from differences in the presentation of annoyance questions and the use of a particular annoyance scale. Even if the isolated influence of any one of the factors investigated here may not be very large, it seems that in sum the combination of several factors is substantial enough (see Figure 6) to challenge previous conclusions about differences in the exposure-response relationships from studies conducted in different “communities” where differences between exposure-response functions are simply explained as differences in the sensitivity of these community populations to noise [21]. It appears plausible that further differences in annoyance responses between studies are also generated by factors such as those which have been studied in our field experiment. More generally, our findings also suggest that between-study methodological differences are so large that conclusions about influencing factors that affect annoyance should only be based on within-study comparisons.

In the present study, we found evidence that early appearance of annoyance questions (early placement in a questionnaire) produces (slightly) higher annoyance ratings. This should be kept in mind when comparing studies that employed different question locations. In order to maximize compatibility and comparability of annoyance ratings across noise studies, we strongly recommend following the original advice by the ICBEN, namely to pose the annoyance questions early in the interview. This probably helps to avoid the creation of a specific frame of reference (context), before annoyance questions are asked, and therefore increases comparability of annoyance ratings between studies. Of course, one may decide to not follow this recommendation if placing noise annoyance questions early would conflict with other objectives of a particular study.

The implicit hypothesis, that reported annoyance tends to be higher in the summertime, could be confirmed in our study. Although survey participants were invited to not pay attention to the current season, but were asked to integrate their noise annoyance experiences over a longer period (12 months), this instruction did not totally remove the season effect. Future researchers should be aware of this potential source of variability when planning a new survey or when pooling survey results, e.g., in meta-analyses.

In order to be able to compare annoyance outcomes to results from previous studies, one may still include both questions/scales in future surveys. As we showed, the sequence of the presentation of the questions does not seem to influence the annoyance rating, nor does the order of response alternatives. The researcher has thus a variety of options as regards the layout/arrangement of ICBEN questions in a questionnaire.

## Figures and Tables

**Table 1:** Factorial stratification including questionnaire return statistics.

Variant	Season	Sequence	Order of Response Alternatives	Location	*n* Returns (Cooperation Rate)
A	Autumn	5-point → 11-point	ascending	early	161 (31%)
B	Autumn	5-point → 11-point	ascending	late	157 (30%)
C	Autumn	5-point → 11-point	descending	early	164 (31%)
D	Autumn	5-point → 11-point	descending	late	142 (27%)
E	Autumn	11-point → 5-point	ascending	early	136 (26%)
F	Autumn	11-point → 5-point	ascending	late	146 (28%)
G	Autumn	11-point → 5-point	descending	early	152 (29%)
H	Autumn	11-point → 5-point	descending	late	136 (26%)
A	Spring	5-point → 11-point	ascending	early	154 (29%)
B	Spring	5-point → 11-point	ascending	late	146 (28%)
C	Spring	5-point → 11-point	descending	early	151 (29%)
D	Spring	5-point → 11-point	descending	late	156 (30%)
E	Spring	11-point → 5-point	ascending	early	135 (26%)
F	Spring	11-point → 5-point	ascending	late	150 (29%)
G	Spring	11-point → 5-point	descending	early	155 (30%)
H	Spring	11-point → 5-point	descending	late	145 (28%)

**Table 2:** Conversions of scale point values on the 11-point numerical and 5-point verbal ICBEN scales to values on an absolute intensity scale ranging from 0 to 100.

**11-Point Numerical Scale and Corresponding Numeric Values on an 0–100 Interval Scale:**											
**Scale Point Label:**	**“0”**	**“1”**	**“2”**	**“3”**	**“4”**	**“5”**	**“6”**	**“7”**	**“8”**	**“9”**	**“10”**
Numeric value:	0	1	2	3	4	5	6	7	8	9	10
Discrete point:	0	10	20	30	40	50	60	70	80	90	100
Midpoint of category:	4.55	13.64	22.73	31.82	40.90	50.00	59.09	68.18	77.27	86.36	95.50
**5-Point Verbal Scale and Corresponding Numeric Values on an 0–100 Interval Scale:**											
**Scale Point Label:**	**“not at all”** **“*iiberhaupt nicht*”**		**“slightly”** **“*etwas*”**		**“moderately”** **“*mittelmässig*”**			**“very”** **“*stark*”**		**“extremely”** **“*äusserst*”**	
Numeric value:	0		1		2			3		4	
Discrete point:	0		25		50			75		100	
Midpoint of category:	10		30		50			70		90	

**Table 3:** Response statistics in both survey waves.

	Wave 1 (Autumn)	Wave 2 (Spring)
Total # persons individually addressed (initial mail order)	4200	4200
Returned non-empty questionnaires	1220	1211
(thereof number of questionnaires with valid addresses)	(1194)	(1192)
Addressee deceased, unable to respond, or language problem ^a^	5	3
Nothing returned	2917	2873
Actively refused by addressee	8	8
Envelope returned undelivered ^b^	50	105
Cooperation Rate	0.29	0.29
Response Rate	0.28	0.28

^a^ Questionnaire sent back empty with explanations by another household member, or addressee called declaring his/her inability to take part in the survey; ^b^ Envelope returned by Swiss Post because of unknown address.

**Table 4:** Sample characteristics (both survey waves pooled).

Ldn ^a^ Category (dB(A))	*n*	% of Sample	% Female	Mean Age (Years)	Mean of Occupancy (Years)	%HA (5-Point)	%HA (11-Point)	MA (5-Point) ^b^	MA (11-Point) ^b^
≤50	15	0.63	60.00	52.20	14.67	6.67	6.67	18.33	26.00
50.0–52.5	49	2.05	57.14	54.18	17.08	8.16	2.04	19.90	20.21
52.5–55.0	183	7.67	56.83	53.03	14.60	12.57	10.38	27.34	28.11
55.0–57.5	247	10.35	55.47	54.28	14.09	9.31	6.48	27.35	29.35
57.5–60.0	282	11.82	52.84	54.34	15.60	11.70	9.93	30.36	31.87
60.0–62.5	370	15.51	54.86	52.73	14.08	18.92	15.14	34.76	37.68
62.5–65.0	373	15.63	54.96	53.48	15.34	21.72	17.96	37.74	40.54
65.0–67.5	426	17.85	46.95	51.79	15.00	28.87	25.59	42.61	45.88
67.5–70.0	278	11.65	58.99	50.74	14.57	26.98	22.66	42.09	45.93
≥70	163	6.83	54.60	52.57	14.77	29.45	23.31	44.33	49.25

^a^ Day–Night Level; ^b^ Original scale values linearly transformed to a 0 to 100 scale using discrete point conversion (see Section 2.3.1); %HA: percentage highly annoyed; MA: Mean annoyance score.

**Table 5:** Average daily weather parameters in both survey waves. The data reflect the mean across individual averaged values of respondents (including standard deviation in brackets), based on weather station closest to respondent’s home.

Wave	Survey Period	Averaging Period ^a^	Day Temp. (°C)	Night Temp. (°C)	Precipitation ^c^ (mm)	Sunshine Hours (h)
Autumn	10 October to 28 November 2012	1 day ^b^	11.14 (3.20)	8.32 (2.80)	2.00 (3.45)	2.89 (2.95)
		7 days	12.48 (2.30)	10.18 (2.13)	4.00 (2.38)	2.38 (1.45)
		30 days	13.89 (1.25)	10.94 (1.02)	2.17 (0.51)	4.16 (0.50)
Spring	11 March to 29 April 2013	1 day ^b^	3.64 (3.69)	1.07 (3.26)	0.98 (1.47)	2.23 (2.47)
		7 days	6.36 (2.85)	3.62 (2.40)	0.69 (0.57)	2.82 (0.94)
		30 days	1.94 (1.13)	0.13 (1.05)	0.30 (0.24)	2.45 (0.40)

^a^ Time period for which the averages are calculated; ^b^ Weather parameters on the day that the questionnaire was filled out; ^c^ Sum in the period 07:00–19:00 h.

**Table 6:** Parameter estimates of a repeated measures mixed model analysis on annoyance score (discrete point conversion).

Effect	Level	*F*	B	SE	*t*	*p* Value
**Between subject effects**						
Intercept		77.831	−44.426	4.875	−9.113	0.000
Noise exposure (Ldn)		277.705	1.272	0.076	16.664	0.000
Season	autumn ^a^	6.491	2.099	0.824	2.548	0.011
Question sequence	5-point → 11-point ^b^	0.121	0.286	0.824	0.347	0.728
Order of response alternatives	ascending ^c^	0.519	0.593	0.824	0.720	0.471
Location in questionnaire	early ^d^	25.758	4.182	0.824	5.075	0.000
**Within subject effect**						
Type of scale	11-point ^e^	11.198	2.763	0.826	3.346	0.001

^a^ Versus spring; ^b^ Versus 11-point → 5-point; ^c^ Versus descending; ^d^ Versus late; ^e^ Versus 5-point; SE: standard error of the estimate.

**Table 7:** Parameter estimates of a repeated measures logistic regression model for the probability of high annoyance (PHA).

Effect	Level	B	95% CI of B		exp(B)	CI of exp(B)		*p* Value
**Between-subject effects**								
Intercept		−7.160	−8.369	−5.952	0.001	0.000	0.003	<0.001
Noise exposure		0.084	0.066	0.103	1.088	1.068	1.109	<0.001
Season	autumn ^a^	0.043	−0.153	0.240	1.044	0.858	1.271	0.665
Question sequence	5-point → 11-point ^b^	−0.019	−0.178	0.216	1.020	0.837	1.242	0.847
Order of response alternatives	ascending ^c^	0.109	−0.087	0.306	1.115	0.916	1.358	0.276
Location in questionnaire	early ^d^	0.248	0.051	0.445	1.281	1.052	1.560	0.014
**Within-subject effect**								
Type of scale	11-point ^e^	−0.236	−0.161	−0.311	1.266	1.175	1.365	<0.001

^a^ Versus spring; ^b^ Versus 11-point → 5-point; ^c^ Versus descending; ^d^ Versus late; ^e^ Versus 5-point.

## Supplementary Materials

The following are available online at www.mdpi.com/1660-4601/13/11/1163/s1, Original questionnaires-zip file containing: Document S1, Questionnaire Variant “A” (in German); Document S2, Questionnaire Variant “A” (English translation); Document S3, Questionnaire Variant “H” (in German); Document S4, Questionnaire Variant “H” (English translation).
