# Supplementary material for: Effects of Scale, Question Location, Order of Response Alternatives, and Season on Self-Reported Noise Annoyance Using ICBEN Scales: A Field Experiment
Source: Int J Environ Res Public Health. 2016 Nov 23;13(11):1163. doi: 10.3390/ijerph13111163 (PMC5129373; doi:10.3390/ijerph13111163)
Supplement: Supplementary file 1 [file ijerph-13-01163-s001.zip › S2 Questionnaire Version A (ENG).pdf]

Short survey on noise in the living environment «A» 《PersID》

Please answer all questions one by one in sequence. Always tick the answer/checkbox that best applies to you. If multiple answers apply, tick all that apply. Thank you very much.

General Information

1.1 Gender: ☐ female ☐ male Year of birth: 11ab

1.2 Is the address in the cover letter
the address where you live most of the time? ☐ yes ☐ no 12a

1.3 How long have you been living in your current home? .... years 13a

1.4 Do you live: ☐ alone ☐ as a family/couple ☐ in a flat share ☐ other 14a

1.5 What kind of dwelling do you live in? ☐ row house ☐ detached house 15a
☐ apartment on ground floor
☐ apartment on .... floor 15b

1.6 Are you the owner or tenant of your apartment/house?
☐ owner ☐ tenant or sub-tenant 16a

Living Environment and Noise

2.1 Thinking about the last 12 months, when you are here at home, how much did road traffic noise bother, disturb, or annoy you?

not at all	slightly	moderately	very	extremely
☐	☐	☐	☐	☐

21a

2.2 Below is a 0 to 10 opinion scale for how much road traffic noise bothered, disturbed or annoyed you. If you were not at all annoyed choose 0, if you were extremely annoyed choose 10, if you were somewhere in between choose a number between 0 and 10.

Thinking about the last 12 months, when your are here at home, what number from 0 to 10 best shows how much you were bothered, disturbed, or annoyed by road traffic noise?

0 1 2 3 4 5 6 7 8 9 10

not at all (0) … extremely (10)

22a

3.1 Below is a list of characteristics that are important for living. How satisfied are you regarding these at your home?

	not at all satisfied	slightly satisfied	modera-tely satisf.	very satisf.	extremely satisf.	
◆ living comfort in general	☐	☐	☐	☐	☐	23a
◆ spaciousness	☐	☐	☐	☐	☐	23b
◆ distances to work / schools etc.	☐	☐	☐	☐	☐	23c
◆ exposure to noise	☐	☐	☐	☐	☐	23d
◆ size of balcony, terrace, or garden	☐	☐	☐	☐	☐	23e
◆ lightness, sunshine	☐	☐	☐	☐	☐	23f

3.2 Is there anything in your home that bothers or disturbs you in particular?
☐ no ☐ yes, that is? ....
.... 24a

please turn ↺

3.3	How often are you disturbed by road traffic noise in the following activities?	
	**never / rarely / some-times / often / always**	
	◆ Disturbs conversations, phone calls etc. ☐ ☐ ☐ ☐ ☐	25a
	◆ Disturbs listening to the radio, watching TV ☐ ☐ ☐ ☐ ☐	25b
	◆ Disturbs recreation outdoors ☐ ☐ ☐ ☐ ☐	25c
	◆ Disturbs reading, thinking, concentration ☐ ☐ ☐ ☐ ☐	25d
	◆ Disturbs sociability, e.g. with guests ☐ ☐ ☐ ☐ ☐	25e
	◆ Disturbs at night, when I want to sleep ☐ ☐ ☐ ☐ ☐	25f
3.4	By which kind of vehicles are you disturbed or annoyed in particular?<br>☐ none ☐ cars ☐ trucks, delivery vehicles ☐ motorcycles ☐ trams/buses	26a
3.5	Are you particularly disturbed or annoyed by road traffic noise at certain times (night or day)?	
	☐ no	
	☐ yes, from _ _ : _ _ to _ _ : _ _ o'clock	27a
	and from _ _ : _ _ to _ _ : _ _ o'clock	27b
	and from _ _ : _ _ to _ _ : _ _ o'clock	27c
4.1	Please indicate how noise sensitive you are in general. Are you...<br>**0 1 2 3 4 5 6 7 8 9 10**<br>**not at all noise sensitive** … **extremely noise sensitive**	31a
5.1	The following questions pertain to your sleeping habits. What is the orientation of your bedroom towards the nearest street?<br>☐ away from street ☐ perpendicular to street ☐ pointing towards street	41a
5.2	Do you have mechanical ventilation (with heat recovery) in your home:<br>☐ yes ☐ no ☐ don't know	42a
5.3	In which position do you keep your bedroom windows usually?	
	◆ in summer: ☐ closed ☐ open or half-open	43a
	◆ in winter: ☐ closed ☐ open or half-open	43b
	if open during summer, why? ☐ fresh air supply ☐ cooling ☐ other	43c
	if closed during summer, why? ☐ noise (from outside) ☐ heat loss ☐ other	43d
	if open during winter, why? ☐ fresh air supply ☐ cooling ☐ other	43e
	if closed during winter, why? ☐ noise (from outside) ☐ heat loss ☐ other	43f
	If it would be totally calm outside (regardless of source) which window position would you prefer? ◆ in summer: ☐ closed ☐ open/half-open	43g
	◆ in winter: ☐ closed ☐ open/half-open	43h
5.4	How often do you use earplugs (e.g. Oropax) for sleeping? **never / rarely / sometimes / often / always** ☐ ☐ ☐ ☐ ☐	44a
	If you use them, why? ☐ because of noise from outside the house	44b
	☐ because of noise from inside the house	44c
	☐ because of noise from within sleeping room (e.g. snoring)	44d
6.1	Today's date: .... ☺ **Thank you for your cooperation!** ☺	
