# Supplementary material for: Effects of Scale, Question Location, Order of Response Alternatives, and Season on Self-Reported Noise Annoyance Using ICBEN Scales: A Field Experiment
Source: Int J Environ Res Public Health. 2016 Nov 23;13(11):1163. doi: 10.3390/ijerph13111163 (PMC5129373; doi:10.3390/ijerph13111163)
Supplement: Supplementary file 1 [file ijerph-13-01163-s001.zip › S3 Questionnaire Version H (GER).pdf]

# Kurz-Umfrage • Lärm im Wohnumfeld • «H» «PersID»

Bitte beantworten Sie die einzelnen Fragen der Reihe nach. Kreuzen Sie jeweils die Antwort an, die auf Sie am ehesten zutrifft. Falls mehrere Antworten zutreffen, kreuzen Sie alle zutreffenden an. Besten Dank.

## Allgemeine Angaben

1.1 Geschlecht: ☐ weiblich ☐ männlich Jahrgang: .................. 11ab

1.2 Ist die Adresse im Begleitbrief der Ort, wo Sie normalerweise wohnen? ☐ ja ☐ nein 12a

1.3 Wie lange wohnen Sie schon in Ihrer jetzigen Wohnung/Haus? .................. Jahre 13a

1.4 Wohnen Sie: ☐ alleine ☐ in einer Familie/als Paar ☐ in einer WG ☐ anderes 14a

1.5 In welcher Art Wohnung/Haus wohnen Sie? ☐ Reihenhaus ☐ Einfamilienhaus 15a
☐ Wohnung im Erdgeschoss
☐ Wohnung im ....... Stock 15b

1.6 Sind Sie Eigentümer/in oder Mieter/in Ihrer Wohnung oder Ihres Hauses?
☐ Eigentümer/in ☐ Mieter/in oder Untermieter/in 16a

## Angaben zum Wohnen und zum Lärm

2.1 Es folgen einige Eigenschaften, die für das Wohnen wichtig sind. Wie zufrieden sind Sie damit bei Ihnen zu Hause?

| | sehr zufrieden | ziemlich zufried. | mittel-mässig | wenig zufried. | nicht zufried. | |
|---|---|---|---|---|---|---|
| ◆ Komfort allgemein | ☐ | ☐ | ☐ | ☐ | ☐ | 23a |
| ◆ Vorhandener Platz, Geräumigkeit | ☐ | ☐ | ☐ | ☐ | ☐ | 23b |
| ◆ Distanz zur Arbeit, Schule etc. | ☐ | ☐ | ☐ | ☐ | ☐ | 23c |
| ◆ Belastungen durch Lärm | ☐ | ☐ | ☐ | ☐ | ☐ | 23d |
| ◆ Grösse von Balkon, Sitzplatz oder Garten | ☐ | ☐ | ☐ | ☐ | ☐ | 23e |
| ◆ Licht und Sonne | ☐ | ☐ | ☐ | ☐ | ☐ | 23f |

2.2 Gibt es in Ihrer Wohnung / Ihrem Haus irgendwelche Dinge, die Sie besonders stören?
☐ nein ☐ ja, welche? .........................................................................................
......................................................................................... 24a

2.3 Wie oft treten bei Ihnen folgende Störungen als Folge von Strassenlärm auf?

| | immer | oft | gelegent-lich | selten | nie | |
|---|---|---|---|---|---|---|
| ◆ Stört bei Unterhaltungen, beim Telefonieren | ☐ | ☐ | ☐ | ☐ | ☐ | 25a |
| ◆ Stört beim Radio-/Musikhören oder Fernsehen | ☐ | ☐ | ☐ | ☐ | ☐ | 25b |
| ◆ Stört bei Aufenthalt und Erholung draussen | ☐ | ☐ | ☐ | ☐ | ☐ | 25c |
| ◆ Stört Lesen, Nachdenken, Konzentration | ☐ | ☐ | ☐ | ☐ | ☐ | 25d |
| ◆ Stört häusliche Geselligkeit, z.B. mit Gästen | ☐ | ☐ | ☐ | ☐ | ☐ | 25e |
| ◆ Stört nachts bzw. wenn ich schlafen will | ☐ | ☐ | ☐ | ☐ | ☐ | 25f |

2.4 Durch welche Fahrzeuge auf der Strasse werden Sie besonders gestört oder belästigt?
☐ keine ☐ Autos ☐ Lastwagen/Lieferwagen ☐ Motorräder ☐ Tram/Bus 26a

2.5 Werden Sie durch Strassenlärm zu bestimmten Zeiten (am Tag oder in der Nacht) besonders gestört oder belästigt? ☐ nein
☐ ja, von _ _ : _ _ bis _ _ : _ _ Uhr 27a
und von _ _ : _ _ bis _ _ : _ _ Uhr 27b
und von _ _ : _ _ bis _ _ : _ _ Uhr 27c

bitte wenden ↺

3.1 Geben Sie bitte auf der folgenden Messlatte an, für wie lärmempfindlich Sie sich ganz allgemein halten. Sind Sie...

10 9 8 7 6 5 4 3 2 1 0

**äusserst lärmempfindlich** … **überhaupt nicht lärmempfindlich** 31a

4.1 Es folgen einige Fragen zum Schlafverhalten. Wie ist die Ausrichtung Ihres Schlafzim mers zur nächstgelegenen Strassse?
☐ von der Strasse weg ☐ seitlich zur Strasse ☐ auf die Strasse gerichtet 41a

4.2 Verfügen Sie in Ihrer Wohnung über eine automatische Lüftung bzw. eine sog. Kom fortlüftung (Minergie-Häuser): ☐ ja ☐ nein ☐ weiss nicht 42a

4.3 In welcher Stellung halten Sie nachts die Schlafzimmerfenster mehrheitlich?
- im Sommer: ☐ zu ☐ offen/halboffen 43a
- im Winter: ☐ zu ☐ offen/halboffen 43b

| | | | | |
|---|---|---|---|---|
| Falls offen im Sommer, warum? | ☐ Frischluftzufuhr | ☐ Abkühlung | ☐ anderes | 43c |
| Falls zu im Sommer, warum? | ☐ Lärm (von aussen) | ☐ Wärmeverlust | ☐ anderes | 43d |
| Falls offen im Winter, warum? | ☐ Frischluftzufuhr | ☐ Abkühlung | ☐ anderes | 43e |
| Falls zu im Winter, warum? | ☐ Lärm (von aussen) | ☐ Wärmeverlust | ☐ anderes | 43f |

Wenn draussen nicht der geringste Lärm (egal welcher Art) wäre, was wäre Ihre bevorzugte Stellung der Schlafzimmerfenster?
- im Sommer: ☐ zu ☐ offen/halboffen 43g
- im Winter: ☐ zu ☐ offen/halboffen 43h

4.4 Wie oft benutzen Sie zum Schlafen Ohrstöpsel oder Ohropax?

**immer** ☐ — **oft** ☐ — **gelegentlich** ☐ — **selten** ☐ — **nie** ☐ 44a

falls ja, warum hauptsächlich?
☐ wegen Geräuschen von aussen 44b
☐ wegen Geräuschen im Haus oder in der Wohnung 44c
☐ wg. Geräuschen im Schlafzimmer (z.B. Schnarchen) 44d

5.1 Hier sehen Sie eine Messlatte von 0 bis 10, auf der Sie angeben können, wie sehr Sie der Strassenlärm insgesamt gestört oder belästigt hat. Wenn Sie sich äusserst gestört oder belästigt fühlten, umkreisen Sie die 10, wenn Sie sich überhaupt nicht gestört oder belästigt fühlten, umkreisen Sie bitte die 0, und wenn Sie irgendwo dazwischenliegen, umkreisen Sie bitte eine Zahl zwischen 0 und 10.

Wenn Sie nun an die letzten zwölf Monate hier bei Ihnen denken, welche Zahl zwischen 0 und 10 gibt am besten an, wie stark Sie sich durch den Strassenlärm insgesamt gestört oder belästigt fühlten?

10 9 8 7 6 5 4 3 2 1 0

**äusserst** … **überhaupt nicht** 22a

5.2 Wenn Sie einmal an die letzten zwölf Monate hier bei Ihnen denken, wie stark haben Sie sich durch Strassenlärm insgesamt gestört oder belästigt gefühlt?

**äusserst** ☐ — **stark** ☐ — **mittelmässig** ☐ — **etwas** ☐ — **überhaupt nicht** ☐ 21a

6. Heutiges Datum: ....................................

☺ **Besten Dank für Ihre Mitarbeit!** ☺
